# Strong Functional Connectivity among Homotopic Brain Areas Is Vital for Motor Control in Unilateral Limb Movement

**DOI:** 10.3389/fnhum.2017.00366

**Published:** 2017-07-12

**Authors:** Pengxu Wei, Zuting Zhang, Zeping Lv, Bin Jing

**Affiliations:** ^1^Beijing Key Laboratory of Rehabilitation Technical Aids for Old-age Disability, Key Laboratory of Neuro-functional Information and Rehabilitation Engineering of the Ministry of Civil Affairs, National Research Center for Rehabilitation Technical Aids Beijing, China; ^2^School of Biomedical Engineering, Capital Medical University Beijing, China

**Keywords:** motor control, functional magnetic resonance imaging, functional connectivity, weighted brain network, graph theory

## Abstract

The mechanism underlying brain region organization for motor control in humans remains poorly understood. In this functional magnetic resonance imaging (fMRI) study, right-handed volunteers were tasked to maintain unilateral foot movements on the right and left sides as consistently as possible. We aimed to identify the similarities and differences between brain motor networks of the two conditions. We recruited 18 right-handed healthy volunteers aged 25 ± 2.3 years and used a whole-body 3T system for magnetic resonance (MR) scanning. Image analysis was performed using SPM8, Conn toolbox and Brain Connectivity Toolbox. We determined a craniocaudally distributed, mirror-symmetrical modular structure. The functional connectivity between homotopic brain areas was generally stronger than the intrahemispheric connections, and such strong connectivity led to the abovementioned modular structure. Our findings indicated that the interhemispheric functional interaction between homotopic brain areas is more intensive than the interaction along the conventional top–down and bottom–up pathways within the brain during unilateral limb movement. The detected strong interhemispheric horizontal functional interaction is an important aspect of motor control but often neglected or underestimated. The strong interhemispheric connectivity may explain the physiological phenomena and effects of promising therapeutic approaches. Further accurate and effective therapeutic methods may be developed on the basis of our findings.

## Introduction

Previous functional magnetic resonance imaging (fMRI) studies have detected several brain regions activated during movement. These regions include the primary motor cortex (M1), supplementary motor area (SMA), dorsal and ventral premotor cortex (PMd and PMv), cingulate motor area (CMA), superior frontal gyrus (SFG), primary and secondary somatosensory areas (S1 and S2, respectively), superior parietal lobule (SPL), inferior parietal cortex (IPC), putamen, insula, thalamus and the cerebellum (Hotz-Boendermaker et al., 2008; Newton et al., 2008; Francis et al., 2009; Trinastic et al., 2010). However, the mechanism by which these brain regions are organized to achieve motor control remains largely unknown (Shadmehr et al., 2010).

Apart from identifying the neural structures that constitute a functional brain system, characterizing the properties of the functional interaction network among brain regions in the brain system is also important (Büchel et al., 1999). Studies on resting-state fMRI have explored the valuable features of brain motor networks, including the specific somatotopy of the functional connections among the primary motor regions during rest (van den Heuvel and Hulshoff Pol, 2010). Nevertheless, the motor network detected during resting state shows different spatial patterns with respect to those of the network detected during motor performance (Kristo et al., 2014). Thus, a brain motor network constructed from resting-state fMRI data may not effectively represent the mechanism behind brain region organization during motor execution.

Studies on task-state functional connectivity are needed to determine whether various types of task-state and resting-state functional connectivities measure similar or different phenomena (Fox and Raichle, 2007). The functional connectivity during resting and task states has been explored in several works (Power et al., 2011; Caeyenberghs et al., 2012). Dynamic changes in the organization of motor learning networks have also been identified by constructing and analyzing task-related networks (Bassett et al., 2011). Compared with motor learning, motor execution is the fundamental form of motor control. Even so, the basic principles of motor execution networks in the normal human brain have not been systematically studied using weighted network analysis. Therefore, these principles must be investigated.

All brain regions involved in a task constitute a network, which is weighed when edges/links among brain regions are assigned with weights. The weight value can be determined using the magnitude of temporal correlation between each pair of regions. A binary network is then transformed from a weighted network but at the cost of losing the weight information of connections (Rubinov and Sporns, 2010).

To determine the general rules of motor execution networks, one must explore the movements performed by limbs on either side of the body. In the current study, we tasked right-handed volunteers to unilaterally move each of their feet in turn over a short duration. The volunteers were also instructed to maintain these right- and left-side movements as consistently as possible. We then examined several network properties of the brain network to control the right foot movement (RightFoot-network, for the dominant limb) or left foot movement (LeftFoot-network, for the non-dominant limb). The similarities between the two networks can help elucidate the mechanism behind brain region organization during movement.

## Materials and Methods

### Subjects

We recruited 18 right-handed healthy volunteers aged 25 ± 2.3 years (20–29 years, nine males). Prior to the experiment, a screening form that included a list of conditions that could endanger a subject’s safety during magnetic resonance imaging (MRI) scanning was signed by each subject.

The experiment was approved by the ethics committee of the Beijing MRI Center for Brain Research. Written informed consent was acquired from each participant. All procedures were performed in accordance with the latest version of the Declaration of Helsinki.

### Motor Task Paradigm

The duration of the fMRI experiment was 396 s for each subject. The experiment comprised 10 rest–task cycles comprising 20 s rest breaks between 16 s movements, except for an initial rest period of 26 s and final rest period of 22 s. The odd-numbered subjects performed left foot movements in odd numbers of task periods and right foot movements in even numbers of task periods. The even-numbered subjects performed the opposite sequence (Supplementary Figure S1).

During each task period, the subjects repetitively performed alternating dorsiflexion (with the range reaching 10°) and foot relaxation. At exactly 4 s prior to each task period, verbal command “ready, right foot, go” or “ready, left foot, go” was delivered for 1.41 s. Movements were paced following an audio cue given every 2 s, and the verbal command “stop” was provided in the last 300 ms of each task period. The audio cues and commands were recorded in advance, presented with the E-Prime software (Psychology Software Tools, Inc., Pittsburgh, PA, USA), and transmitted via the magnetic resonance (MR) scanner’s intercom system.

Each subject was trained for 5 min prior to the MR scanning. The subjects were requested to keep their hip and knee joints motionless and perform their right- and left-foot movements as consistently as possible. A video monitor during MR scanning was used to observe the motor performance of each subject.

### MRI Data Collection

We used a whole-body 3T Siemens Magnetom Trio system for MR scanning. The equipment was located at the State Key Laboratory of Brain and Cognitive Science at the Beijing MRI Center for Brain Research. During scanning, the subjects lay supine with their eyes closed. The duration of the fMRI experiment included a 4 s lead-in period. Gradient echo images with blood oxygen level dependent (BOLD) contrasts were collected (TR = 2000 ms, TE = 30 ms, flip angle = 75°, field of view = 200 mm × 200 mm, matrix size = 64 × 64). A total of 32 axial slices of 4 mm slice thickness were acquired. T1-weighted images were attained with a 3D magnetization-prepared rapid gradient-echo sequence with a voxel size of 1 mm × 1 mm × 1 mm.

### Preprocessing

Image analysis was performed using the software SPM8 (Wellcome Department of Imaging Neuroscience, University College London, UK). The functional images were first motion corrected. Data were excluded if the head movements exceeded 1 mm/1° (translation/rotation) on any axis. The T1 image was then co-registered to the mean of the realigned functional images. The co-registered T1 and functional images were transformed into the Montreal Neurological Institute (MNI) space and resampled to a voxel size of 3.0 mm × 3.0 mm × 3.0 mm. The functional images were spatially smoothed using a 6 mm full width at half-maximum Gaussian kernel.

### Statistical Analysis

Statistical analyses of BOLD signal changes were performed at two levels using the general linear model. Each condition (task vs. rest) was modeled using a boxcar function convolved with the hemodynamic response function. Regressors of interest (i.e., right foot movement and left foot movement) were modeled as a boxcar function with a length of 16 s convolved with the canonical hemodynamic response function. A high-pass filter of 128 s was applied to remove low-frequency noise. In group analysis, significant signal intensity changes in each condition were identified using the mixed-effects model. The threshold was set at *P* < 0.05, false discovery rate (FDR) corrected for multiple comparisons over the entire brain with a minimum cluster size of 10 voxels.

The brain activation locations were defined using the SPM Anatomy Toolbox (Eickhoff et al., 2005). When no corresponding probabilistic cytoarchitectonic map for a brain region was available in this toolbox, the Human Motor Area Template (for SMA, PMd and PMv; Mayka et al., 2006), the Basal Ganglia Human Area Template (for caudate nucleus and putamen; Spraker et al., 2010), or the WFU Pick Atlas (for cingulum, anterior insula, SFG and thalamus) was used (Maldjian et al., 2003).

### Network Analysis

We used peak activations evoked by unilateral foot movement to define the node centers in the network construction (a node represents a brain region) of foot movement on the contralateral side. This arrangement was adopted because the determined regions would be partially affected by data noise and the subsequent network analysis would be biased if the activated clusters of right foot movement were used to define the nodes of the RightFoot-network (Kriegeskorte et al., 2009). In the present study, noise refers to the signals that do not include the experimental effects in question. We used a sphere of 3 mm radius for all nodes, as applied in a previous study (Lindner et al., 2010). Together, neither the node centers nor scopes were derived from the activation analysis.

Functional connectivity, which is defined as the temporal correlations between spatially remote neurophysiological events, was estimated with the Conn toolbox designed for resting-state and block data (Whitfield-Gabrieli et al., 2009; Gabitov et al., 2016). The waveform of each brain voxel was filtered using a bandpass filter (0.008 < *f* < 0.09) to reduce the effect of low-frequency drift and high-frequency noise. Six parameters obtained by rigid-body head motion correction (three-rotation and three-translation parameters) were defined as first-level covariates. The signals from ventricular regions, white matter, and their temporal derivatives were also removed by linear regression. Block regressors were convolved with the canonical hemodynamic response function to account for the hemodynamic delay. The correlation coefficient is an easily interpreted metric (Schoppe et al., 2016). For each task, Pearson’s correlation coefficients between BOLD time courses from each node pair were computed. Then, the coefficients were converted to normally distributed scores by using Fisher’s transform under a second-level random-effect analysis. The threshold of the correlation magnitudes was *P* < 0.05 and FDR corrected for multiple comparisons. A correlation’s magnitude was used as the weight of an edge/link upon passing such threshold.

We determined the distance *D* between two brain regions by using *D* = 1 − *W*, as described in a previous study (Achard and Bullmore, 2007). *W* is the weight of the link between the two regions. All network features were calculated using the Brain Connectivity Toolbox based on graph theory (Rubinov and Sporns, 2010). Networks were visualized with the BrainNet Viewer (Xia et al., 2013).

## Results

### Brain Activations

**Figure 1:**
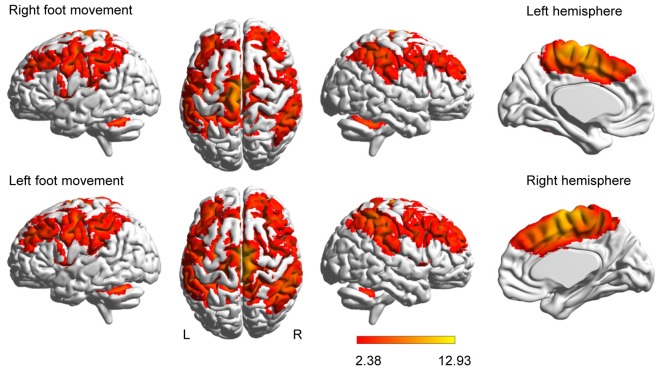
Brain activation evoked by movement. Activations evoked by foot movement are projected onto the normalized 3D brain with the BrainNet Viewer (http://www.nitrc.org/projects/bnv/).The last column of each row shows a midsagittal view of the hemisphere contralateral to the moved foot. The color bar shows the *t* values.

Brain activation was more extensive during the left-foot movement than during the right-foot movement (11,085 voxels vs. 9695 voxels, respectively), as revealed by the group-level analysis of 18 subjects (Figure 1). This result is consistent with that of a previous study (Ciccarelli et al., 2005).

Besides the four unilateral areas, namely, the contralateral M1, S1, the thalamus and the ipsilateral cerebellar vermis, the right- or left-foot movement additionally elicited 12 pairs of homotopic areas, namely, SMA, CMA, PMd, PMv, SFG, SPL, IPC, S2, anterior insula, putamen, caudate nucleus and cerebellar hemisphere (peaks shown in Supplementary Table S1). Generally, the contralateral peak *T* values were higher than the ipsilateral *T* values.

### Basic Network Properties

Each network consisted of 28 brain regions, with 12 pairs and four unilateral regions. Both networks only contained positive connections. A fully connected network of *n* brain regions comprises 1/2 *n* (*n* − 1) connections (378 connections for 28 regions). In particular, the RightFoot-network and LeftFoot-network contained 144 and 95 connections, respectively. Results on the small-world properties, strength, assortativity, betweenness centrality and global efficiency are presented in the Supplementary Material.

### Modular Structure

**Figure 2:**
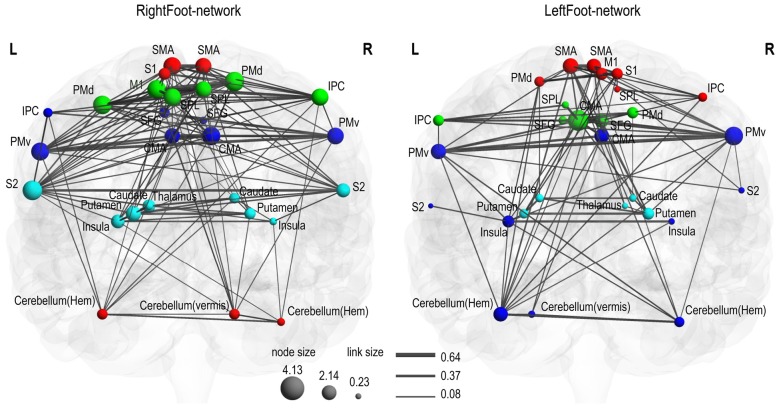
Motor control networks in anatomical space. Two networks are visualized with the BrainNet Viewer in coronal view. Brain regions are sized according to strength (i.e., the sum of the weights of all links connected to this region), and links are sized according to its weight. Brain regions in the same module are indicated in the same color. In the RightFoot-network, the caudate and thalamus are overlapped, but the strength of the thalamus is lower and thus it is smaller in size. For each network, the modules spatially distribute along the cranial–caudal direction and the brain regions in each module show a symmetrically distributed tendency against the midsagittal plane.

Modules are groups of densely interconnected brain regions that are only sparsely connected to the rest of the network. Thus, brain regions within a module achieve a relatively fast information transmission rate, and different modules perform different functions with some degree of independence (Bassett et al., 2011). As shown in Figure 2, both the RightFoot-network and the LeftFoot-network consisted of four modules.

**Figure 3:**
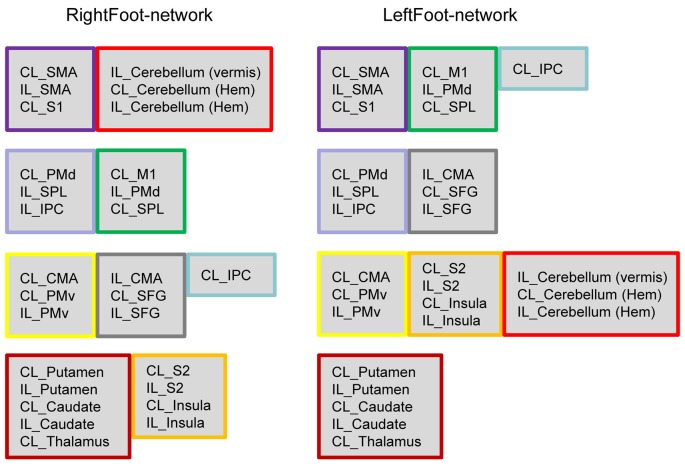
Brain regions in each module. Both networks comprise four modules, which are shown in four rows. If several brain areas in a module in either network appeared together in a module in another network, this group of areas is framed in the same color. The brain areas in the modules exhibit a “symmetric” pattern. In the LeftFoot-network, the ratios between the symmetric brain areas and all areas in each module are 2/7, 2/6, 8/10 and 4/5, the total of which is 16/28. In the RightFoot-network, the ratios are 4/6, 4/6, 6/7 and 8/9 in each module; the total is 22/28. The RightFoot-network obviously shows a more symmetric pattern than the LeftFoot-network.

Most homotopic brain areas in the LeftFoot-network (8 out of 12 pairs, excluding PMds, SPLs, IPCs and CMAs) and in the RightFoot-network (11 out of 12 pairs, excluding IPCs) were contained in pairs in a module. A single module contained at least one pair of homotopic areas for the LeftFoot-network or at least two pairs for the RightFoot-network. The module with the most number of brain areas contained the maximum number of such pairs for both networks. The largest module in the LeftFoot-network contained 10 brain regions including four pairs of homotopic brain areas, and the largest module in the RightFoot-network contained nine brain regions including four pairs of homotopic areas (Figure 3).

The probability that most homotopic brain areas were contained in pairs in a module was very low for each network (Supplementary Text S1). Thus, the interaction between the homotopic brain regions was a prominent feature in the motor control networks at the module level, other than an event observed by chance.

Given that most of the homotopic areas tended to operate collectively in modules, the brain areas in the modules exhibited symmetric patterns to various extents. Particularly, the RightFoot-network showed this tendency to a greater degree than that of the LeftFoot-network (Figure 2).

**Figure 4:**
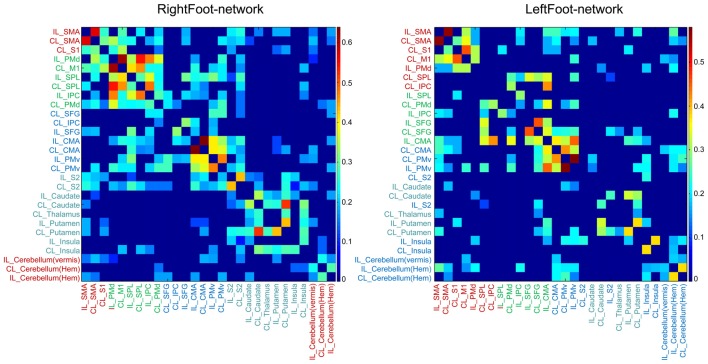
Matrix of link weights. The colored bars indicate the Fisher-transformed correlation coefficient values. Brain regions are sequenced along the cranial–caudal direction according to the *z* values of the MNI coordinates. Brain regions in the same module are shown in the same color. Note that the modular structure is consistent with such a sequence for both networks.

The modules in the RightFoot-network were distributed along the cranial–caudal direction (Figure 2). The areas at the topmost part of the brain constituted a module and then those located caudally formed the next. Such process continued until the basal areas of the cerebral hemispheres constituted the fourth module. The cerebellar areas were part of the topmost module. The modules in the LeftFoot-network showed a similar spatial pattern. This spatial pattern in modularity was further confirmed when we listed all the brain regions in a network along the cranial–caudal direction in a matrix of connection weights (Figure 4).

### Distribution of Strongest Links

In a weighted network, each link carried a numerical value corresponding to the link’s weight. The link weight of functional connectivity between certain brain regions is related to cognitive function in patients with mild cognitive impairments (Liu et al., 2014). This finding emphasizes the physiological/pathophysiological significance of the link weight between brain regions.

**Figure 5:**
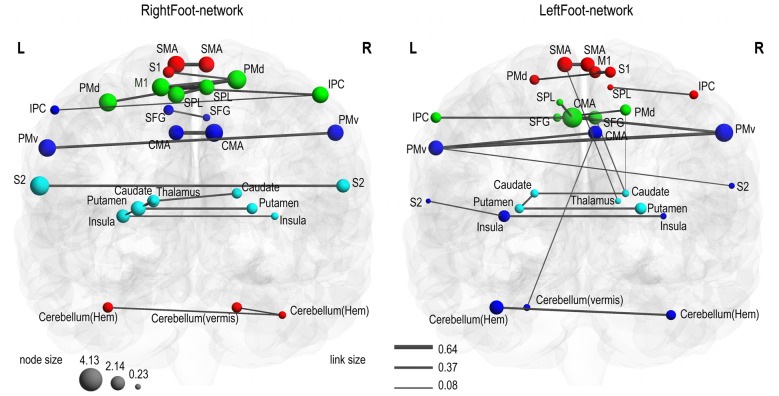
Strongest connections. In each network, the strongest link connecting each brain area is shown. Most symmetric brain regions were connected to each other through the strongest links. In the RightFoot-network, 18 out of the 24 symmetric areas were connected by such links; in the LeftFoot-network, 13 out of the 24 symmetric areas were connected by such links.

In each network, the link with the highest weight among all the links connecting each brain region was selected, and 28 of the strongest links were finally acquired. Most of these 28 links connected the homotopic brain regions (Figure 5). Thus, the two motor control networks exhibited consistent results. However, the RightFoot-network exhibited a more extensive distribution of the strongest links between homotopic brain areas than that of the LeftFoot-network.

This phenomenon of homotopic brain regions being connected by the majority of the 28 strongest links for each network was unlikely observed by chance, a finding confirmed by the low probability values (Supplementary Text S2).

## Discussion

Similar to previous reports (Francis et al., 2009; Trinastic et al., 2010), unilateral foot movement activated many homotopic brain regions in our study. Contralateral peak *T* values were also higher than the ipsilateral *T* values. This result is consistent with the conventional concept that unilateral limb movement is mainly controlled by the contralateral hemisphere.

### Cranial–Caudally Distributed, Mirror- Symmetrical Modular Structure

Modules are groups of densely interconnected brain regions in a network; only sparser links exist between modules. Thus, different modules can perform different functions with some degree of independence (Newman, 2006). We found the following two features in the modular structure: (1) The modules in the two networks were distributed along the cranial–caudal direction. That is, most brain areas in each module were located in the same segment along the cranial–caudal direction. (2) No module contained brain areas from a unilateral hemisphere only; each module consisted of brain areas from bilateral hemispheres. Most of these brain areas were symmetrically distributed in two hemispheres, especially in the RightFoot-network. To date, such a craniocaudally distributed, mirror-symmetrical modular structure has not been reported for motor control networks in the human brain.

This finding indicates that brain areas in the same segment and from bilateral hemispheres tend to be connected closely during motor execution. The distribution of the strongest links crucially explains this pattern (most of the 28 strongest links connected the homotopic brain regions in both networks). That is, in a weighted network, the weight and number of links connecting each brain area influence the modularity. As a result, the homotopic areas connected by the strongest links in this study tended to form a module.

### Interhemispheric Horizontal Functional Interaction during Motor Control

Strong functional connectivity between homotopic brain regions is a prominent feature of motor control. This attribute was reflected by both the modular structure and the distribution of the strongest links in the two motor control networks. In both aspects, the RightFoot-network showed a tendency to a greater degree, i.e., more extensive and stronger functional connectivity between homotopic brain areas than that of the LeftFoot-network.

Temporal correlations indicate a cooperative relationship between pre- and postsynaptic neurons (Singer, 1990). Therefore, the temporal correlations detected in this study suggest a similar relationship between homotopic brain areas.

The possible anatomical explanations for this finding are as follows. Although the majority of motor pathways are contralateral, some descend into the spinal cord ipsilaterally (Zaaimi et al., 2012). Thus, limbs on one side are controlled by the motor cortex in both hemispheres. The cooperation between homotopic brain motor areas may also harmonize their actions to ensure coordination at the central level. Similarly, a part of the sensory pathways is ipsilateral (Murphy and Corbett, 2009). Thus, cooperation between homotopic sensory areas is required to process sensory input from unilateral limbs.

One hemisphere may also control certain aspects of movement to a greater degree than could the other hemisphere. When using their ipsilesional arm, patients with left hemisphere damage cannot properly control their arm’s trajectory because of impaired coordination between multiple joints. By contrast, patients with right hemisphere damage show deficits in final position accuracy but with intact multiple joint coordination (Schaefer et al., 2009). Such hemisphere-dependent advantages can be integrated through the cooperation between homotopic brain areas.

### Interhemispheric Connectivity vs. Intrahemispheric Connectivity

Unilateral limb movement is believed to be mainly controlled by the contralateral hemisphere (Rizzolatti and Kalaska, 2013). Thus, we expect that functional interaction during unilateral limb movement should propagate mainly along pathways within the contralateral hemisphere. This pattern should lead to the strongest links between intrahemispheric brain areas in the brain network, i.e., strongest intrahemispheric functional connections between brain areas in the contralateral hemisphere. For example, intrahemispheric links (e.g., between M1, S1, SMA, the thalamus and putamen in the hemisphere contralateral to the moved foot) should be stronger than the interhemispheric links between brain areas during motor control, and the modular structure may exhibit a lateralized pattern (e.g., one or more modules consist of brain areas mainly or only in the hemisphere contralateral to the moved foot) instead of a symmetric pattern.

However, our findings significantly differ from these expectations. Instead, most of the homotopic regions in the brain motor networks were connected to one another through the strongest links. Specifically, the interhemispheric functional interaction between homotopic brain areas was generally more intensive than the functional connectivity along the traditional top–down and bottom–up pathways within the contralateral hemisphere during motor execution, especially for the RightFoot-network. Obviously, such strong inter-hemispheric horizontal functional connectivity is a prominent feature of motor control for unilateral limb movement.

### Rationale Behind the Strong Functional Connectivity between Homotopic Brain Regions

First, the strong functional connectivity may be a basic need of bilateral limb movement. Bilateral movements, such as walking, are common behaviors for bipedal and tetrapod vertebrates. The muscle activities of one arm may also be closely related to the motor actions performed by another arm (Taylor, 2005). Thus, homotopic brain regions in the two hemispheres should closely interact with one another to coordinate the movements performed by both sides. Such close interactions between homotopic brain regions are preserved during unilateral limb movement.

Another benefit is that these strong connections can retain two information copies in each of the two homotopic brain areas. This redundancy reduces the vulnerability of the whole network. That is, unilateral brain damage will be compensated by the contralateral brain regions. Indeed, several studies have shown bilateral brain activation during the recovery phase of stroke rehabilitation (Favre et al., 2014).

The strong functional connectivity between homotopic brain regions during unilateral limb movement also provides a neural basis for the interlimb transfer of motor skills. If unilateral limb movement is only (or mainly) controlled by the contralateral hemisphere and only one in each pair of homotopic brain areas participate in motor control, a person must learn a unilateral motor skill from the beginning even after acquiring the skill through the contralateral side. Instead, given the strong connections between homotopic brain areas, two hemispheres participate in unilateral motor learning and motor control process and are thus both “familiar” with the task. Therefore, a person can master a unilateral motor skill more easily if he or she has already performed the task thoroughly with the other side. Specifically, the detected strong interhemispheric connections can facilitate the motor learning process.

### Physiological Phenomena and Therapeutic Effects Explained by the Findings

Our findings can explain physiological phenomena and the effects of promising therapeutic approaches, such as the unilateral strength training for contralateral improvement (Carroll et al., 2006), bilateral movement training for improving motor performance in the affected limb (Cauraugh et al., 2010), cross education (Ruddy and Carson, 2013; Ruddy et al., 2016), and mirror therapy (Thieme et al., 2013). All these methods apply unilateral or bilateral motor training to improve the motor function of the contralateral side. The underlying mechanisms of these approaches remain controversial, but substantial overlap between the neural processes underlying bilateral and unilateral movements has been proposed (Wang et al., 2013). Currently, the strong interhemispheric functional connectivity during unilateral limb movement provides an intuitive explanation.

Developing neurorehabilitation technologies requires the profound understanding of the mechanisms on motor control (Sartori et al., 2016). Given our findings, further effective therapeutic methods may also be developed.

In this study, we examined the brain activation and network properties of right- or left-foot movement. The applied task is a basic form of motor control, i.e., motor execution. During unilateral limb movement, interhemispheric horizontal functional connectivity was found to be more intensive than the interaction along the conventional top–down and bottom–up pathways within the brain. However, compared with fMRI, electroencephalography and functional near-infrared spectroscopy possess higher temporal resolutions and must hence be applied for further research.

Many previous studies, such as those of Francis et al. (2009) and Trinastic et al. (2010), found that unilateral foot movement activates several homotopic brain regions. Given these findings, we applied weighted network analysis with graph theory to determine the mechanism of brain region organization during motor control. However, we only examined the network properties for a certain foot movement type. Whether other movement types of the foot and other body parts follow the principles revealed in our study remains to be investigated. For instance, some published studies employing finger-tapping tasks consistently observed activations in bilateral sensorimotor cortices, bilateral inferior parietal cortices, bilateral basal ganglia and bilateral anterior cerebellum (Witt et al., 2008). However, whether the strongest functional connections exist between these homotopic brain areas has not been explored.

## Author Contributions

PW, ZZ and ZL conducted the experiments, PW and BJ analyzed the data, PW designed the work, all authors wrote the article.

## Conflict of Interest Statement

The authors declare that the research was conducted in the absence of any commercial or financial relationships that could be construed as a potential conflict of interest.
