# Supplementary material for: Strong Functional Connectivity among Homotopic Brain Areas Is Vital for Motor Control in Unilateral Limb Movement
Source: Front Hum Neurosci. 2017 Jul 12;11:366. doi: 10.3389/fnhum.2017.00366 (PMC5506200; doi:10.3389/fnhum.2017.00366)
Supplement: Supplementary file 1 [file Presentation_1.pdf]

# *Supplementary Material*

# Strong Functional Connectivity among Homotopic Brain Areas is Vital for Motor Control in Unilateral Limb Movement

**Pengxu Wei[1]*, Zuting Zhang[1], Zeping Lv[1], Bin Jing[2]**

*** Correspondence:** Corresponding Author: pengxuwei@gmail.com

## 1 Supplementary Data

### Text. S1 Probability of most homotopic brain areas contained in a module in pairs.

Most of the homotopic brain areas in the LeftFoot-network (8 out of 12 pairs, excluding PMds, SPLs, IPCs, and CMAs) and the RightFoot-network (11 out of 12 pairs, excluding IPCs) were contained in a module in pairs. A single module contained at least one pair of homotopic areas for the LeftFoot-network or at least two pairs for the RightFoot-network. The module with the maximum number of nodes contained the maximum number of such pairs for both networks. The largest module in the LeftFoot-network contained 10 nodes and 4 pairs of homotopic brain areas, and the largest module in the RightFoot-network contained 9 nodes and 4 pairs of homotopic areas.

The probability of such events was very low for each network. For the LeftFoot-network containing 28 brain regions, the maximum probability of a module with 10 brain regions containing 4 pairs of homotopic regions was $9/27 \times 7/25 \times 5/23 \times 3/21$, i.e., $< 0.003$ (the chance that 1 of the 10 regions had its homotopic counterpart in this module was 9/27; the chance that another region had its homotopic counterpart in this module was 7/25 at most when one pair of homotopic areas existed in this module; the chance that the next brain region had its homotopic counterpart in this module was 5/23 at most when two pairs of homotopic areas existed in this module; and the chance that the fourth brain region had its homotopic counterpart in this module was 3/21 at most when three pairs of homotopic areas existed in this module). The overall probability was even lower because of other constraints, such as at least one pair of homotopic areas existing in a single module. Similarly, the maximum probability of such events for the RightFoot-network was $8/27 \times 6/25 \times 4/23 \times 2/21$, i.e., $< 0.001$.

### Text. S2 Probability of most strongest connections linking symmetric brain regions in a network.

Most symmetric brain regions were linked to each other through the strongest connections. In the RightFoot-network, 18 out of the 24 symmetric areas were linked by such connections (ipsilateral SMA, contralateral SMA, ipsilateral CMA, contralateral CMA, contralateral PMd, ipsilateral PMv, contralateral PMv, ipsilateral SFG, contralateral SFG, contralateral SPL, contralateral IPC, ipsilateral S2, contralateral S2, ipsilateral insula, ipsilateral putamen, ipsilateral caudate, ipsilateral cerebellar hemisphere, and contralateral cerebellar hemisphere). In the LeftFoot-network, 13 out of the 24

homotopic areas were linked by such connections (ipsilateral SMA, contralateral SMA, ipsilateral PMv, contralateral PMv, ipsilateral SFG, contralateral SFG, ipsilateral insula, contralateral insula, ipsilateral putamen, contralateral putamen, contralateral caudate, ipsilateral cerebellar hemisphere, and contralateral cerebellar hemisphere).

In a brain network, the degree of brain region *i* is the number of connections linked to this region. The chance *P* of a region *i* with a degree $D_i$ to connect another region with its strongest connection is $1/D_i$. For the RightFoot-network, if the strongest connection of each region was randomly distributed in all its connections, the probability of the 18 symmetric brain regions being simultaneously linked by their strongest connections was the arithmetic product of all related $P_i$ ($1/D_i$). The maximum probability value was $7.9877 \times 10^{-17}$ when considering the 18 lowest degree values in the RightFoot-network. Similarly, the maximum probability value for the LeftFoot-network was $3.4446 \times 10^{-7}$ when considering the 13 lowest degree values in the LeftFoot-network. Such low probability values indicated that the finding (for each network, most of the 28 strongest connections linked the symmetric brain regions) was unlikely observed by chance.

Note that the strongest connection linking a pair of homotopic areas did not necessarily mean that this connection was the strongest one for both areas. For instance, for the three areas $A_L$, $A_R$, and B in a network, connection $A_L$–$A_R$ was the strongest one linking area $A_L$ among all its connections, whereas the connection $A_R$–B might be the strongest connection among all the connections of area $A_R$.

## *Methodological Considerations*

***Network construction.*** Choosing certain brain regions as nodes in a graph and then determining the functional connectivity (edge) between each pair of nodes were required during the network analysis. The results of network analyses may be heavily influenced by approaches to determine the scope and location of nodes (Wang et al., 2009; Zalesky, et al., 2010). Nodes may comprise all brain regions parcellated on the basis of an atlas (Bassett et al., 2011) or only certain brain regions often identified by activations evoked by tasks (Wang et al., 2010). All brain regions participating in motor control should ideally be contained in the graph, and those uninvolved should be excluded. Thus, the results of the activation analysis performed in the current work were used to determine which brain regions should be included in the constructed networks to avoid bias from arbitrary selection.

Our pre-arranged choice was to use peak locations reported by previous studies on foot movement as node centers. However, because spatial distributions of activation evoked by a right or left foot movement are basically mirror-symmetric (see *Results* Section), we used the brain activations evoked by the foot movement on one side to define the node centers in the network construction of foot movement on the contralateral side to bypass circular analysis (Kriegeskorte et al., 2009). For instance, the peak points of brain activations evoked by the right foot movement were flipped against the midsagittal plane and then used as node centers for the LeftFoot-network. The requirements of foot movement on both sides were consistent in the current study; thus, choosing the nodes on the basis of the current approach and not on the basis of a pre-arranged choice could prevent large deviations in node centers caused by inconsistencies in the detailed requirements of foot movement across different studies.

The mean value of signal changes across all voxels in a brain region may not adequately represent such region because brain activations are often detected in some but not all voxels within a brain region. In addition, a large node may extend beyond the scope of a small brain region, such as a caudate nucleus. Therefore, we used a relatively small scope with a radius of 3 mm for all nodes, as used in a previous study (Lindner et al., 2010).

***Detecting the modular structure.*** To detect the modular structure for each network, the algorithm was repeated for 100 times, and the 100 results were found to be identical. These results indicated that the two detected solutions for the RightFoot-network and LeftFoot-network were stable. The Q values of the RightFoot-network and LeftFoot-network were 0.2829 and 0.3345, respectively.

The obtained modules should be compared with the degree-, weight-, and strength-preserving null models containing the same number of nodes (Bullmore and Sporns, 2009). For each network, 100 null models were generated with the Brain Connectivity Toolbox. Subsequently, 100 solutions for modularity detection were calculated for each constructed null model. The solution with the highest correlation coefficient value relative to the original solution was then identified as the representative of the 100 results because such solution was the closest to the original solution. Thus, 100 representatives from the 100 constructed null models were obtained. For the RightFoot-network or LeftFoot-network, the correlation coefficient values between the original solution and its corresponding 100 representatives were $0.21 \pm 0.13$ (mean ± SD) or $0.21 \pm 0.11$ (mean ± SD), respectively. The low correlation coefficient values indicated that the solutions acquired from the two original networks were not associated with the weight, degree, and strength properties of the two networks (Rubinov and Sporns, 2011).

***Changes in brain organizational pattern to meet functional needs.*** A resting-state fMRI study found that two biological factors, the distance between nodes and the common input shared by neighboring nodes, could heavily influence the topological properties of functional brain networks (Vertes et al., 2012). Nevertheless, the differences between the two networks constructed from our task-state data indicate that brain status is also important in the topological properties of functional brain networks because the two factors (distance and input) were consistent in the two networks, whereas about a half of the nodes in each module changed. In other words, the brain can alter its organizational pattern to meet functional needs, which can only be observed when functional brain networks under different statuses are compared. Conversely, the similarities in several network measures between the two constructed networks suggest a certain degree of stability in the brain organizational pattern, which should be caused by biological and anatomical constraints, such as the aforementioned distance and input.

***Considerations of network comparison.*** We modified a motor task paradigm applied in many studies (Sahyoun et al., 2004; Dimitrova et al., 2006; Kapreli et al., 2007; Hotz-Boendermaker et al., 2008; Newton et al., 2008; Francis et al., 2009; Trinastic et al., 2010) and detected brain activation in accordance with the aforementioned reports in the literature. This outcome provided a good basis for our network analysis.

To elucidate the mechanism behind the organization of brain regions during movement, we used task-state fMRI data in constructing weighted functional brain networks. Brain networks detected from resting-state fMRI data (Achard et al., 2006; Wang et al., 2009) contain dozens of brain regions (e.g., 70 or 90). In the current study, although the constructed networks comprised only less than 30 brain regions, those areas most commonly involved in motor control were included. Such

"simplified" networks may intuitively exhibit properties of motor control networks and may thus lead to feasible physiological explanations for our findings.

Various problems are involved in comparing functional brain networks. For weighted networks, unequal numbers of nodes and edges, different weights of corresponding edges, and various thresholds for edge detection between graphs can bias the results of comparisons (Stam et al., 2014). A fully unbiased comparison may be difficult to accomplish, but good approaches perform better than others (van Wijk et al., 2010).

In the current study, we balanced the abovementioned factors between the constructed networks as far as possible to identify the similarities and differences between the two networks in a straightforward way. A previous study found that movements performed with lower limb joints are characterized by a weaker lateralized pattern of brain activation in comparison with hand movements (Kapreli et al., 2006); this result suggests that foot movements may be the appropriate choice for comparing unilateral limb movements. The RightFoot-network and LeftFoot-network comprised the same number of nodes. Of all the 28 nodes in each network, 12 pairs were nearly symmetrically located. In addition, the four unilateral nodes in one network were basically mirror symmetric to those in the other network. This symmetry led to the consistency in the spatial locations of the nodes in the two networks. Such anatomical consistency resulted in similar distances in the anatomical space between each corresponding pair of nodes and in similar common inputs shared by each corresponding node in the two networks; these two factors, the distance and input, have a significant effect on the topological properties of functional brain networks (Vertes et al., 2012).

We also used a consistent threshold for edge detection. Selecting distinct thresholds for different networks to match the sparsity is not appropriate, whereas different edge numbers produced at the same threshold may result in detected differences in the topological or organizational properties between networks (Zalesky et al., 2010). Nevertheless, we found that the RightFoot-network largely reserved its network properties in many aspects when the connection density was reduced. Thus, the unmatched sparsity between the RightFoot-network and the LeftFoot-network unlikely played a major role in the detected differences in network measures. Moreover, a lower sparsity of the RightFoot-network in itself is one of the inherent features of this graph after maintaining a good baseline as far as possible.

***Classification of activated brain areas.*** Distinct regions of prefrontal cortex mediate various functions (Koenigs et al., 2008; Koenigs and Grafman, 2009). Previous studies (Johannsen et al., 2001; Sahyoun et al., 2004; Newton et al., 2008; Trinastic et al., 2010) reported SFG activation evoked by foot movement. SFG activation is considered to be associated with functions related to self-awareness (Goldberg et al., 2006). In the motor task of the current study, the volunteers were tasked to perceive the amplitude, speed, and symmetry of foot movement with their eyes closed. Such a condition may be involved in self-awareness related functions and thus elicit SFG activation.

Several studies (Binkofski et al., 2001; Bohlhalter et al., 2002; Hartmann et al., 2008) suggested that SPL plays a role in the specific processing of perceived kinesthetic inputs during action-related somatosensory information processing. These studies focused on the interactions between hand movement and simultaneous tactile information processing. We proposed a similar role of SPL activation detected in the current work because the motor task we applied here similarly involved processing somatosensory (proprioceptive) inputs during movement.

Results from a number of studies (Hua et al., 2005; Bjornsdotter et al., 2009; Henderson et al., 2011) indicate a rough somatotopy of the human body in the posterior insula. Nevertheless, in the current study, we detected bilateral insular activations only in the anterior–dorsal region. A meta-analysis revealed that attention, language, or memory processes can evoke such insular activation patterns, among a number of cognitive, emotional, motor, and sensory tasks (Viviani et al., 1998). We requested each subject to move his/her foot following the audio cue and maintain foot movements between the right and the left sides as consistently as possible; each subject had to pay close attention to follow these requirements. Thus, we proposed that attention processing was the reason for the insular activations.

Our results show that unilateral foot movement evokes three activated clusters in the cerebellum. These clusters are located in the ipsilateral vermis and in the bilateral cerebellar hemisphere. A previous study demonstrated that the cerebellar vermis is activated by the control of foot movement *per se* [refer to Fig. 7 in (Grodd et al., 2001) for the functional somatotopy for movements of the lips, tongue, hands, and feet in the cerebellum]. Another study found that the activations in the lateral cerebellum are related to sensory processing (Gao et al., 1996).

***Small-world properties.*** The weighted clustering coefficient $C$ and weighted characteristic path length $L$ of each network were scaled with the mean clustering coefficient $C_{\text{random}}$ values and mean characteristic path length $L_{\text{random}}$ values of 100 generated random networks. In the interval of sparsity (or the number of existing edges over the maximum possible number of edges) from 144/378 (RightFoot-network) to 95/378 (LeftFoot-network), the result for the RightFoot-network ($L/L_{\text{random}} = 1.06$ and $C/C_{\text{random}} = 1.24$) and that for the LeftFoot-network ($L/L_{\text{random}} = 1.05$ and $C/C_{\text{random}} = 1.38$) demonstrated a mild tendency toward small-world organization.

Although the numbers of nodes and the approaches to define nodes were relatively different, these results are consistent with a resting-state fMRI study in terms of the interval of the connection densities of the two networks [see Fig. 1A in (Wang et al., 2009)]. This outcome indicates that motor control networks detected during task state and resting state can share similar features for certain network properties.

# 2 Supplementary Figures and Tables

## 2.1 Supplementary Figures

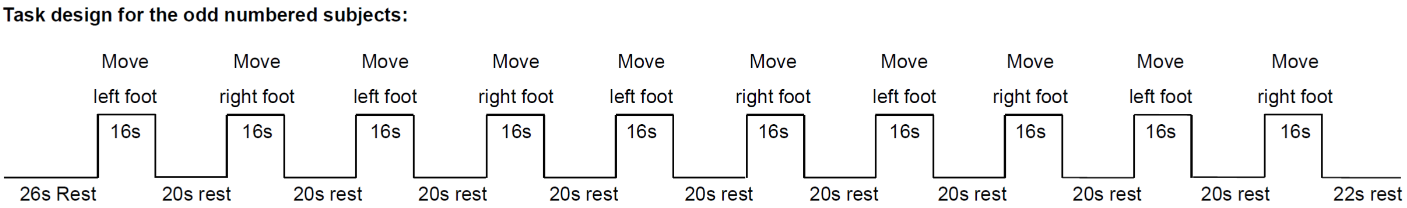


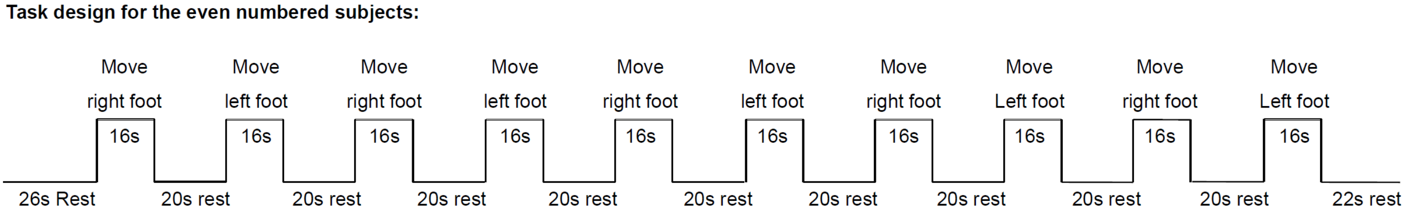


**Supplementary Figure 1. The paradigm of motor task.**

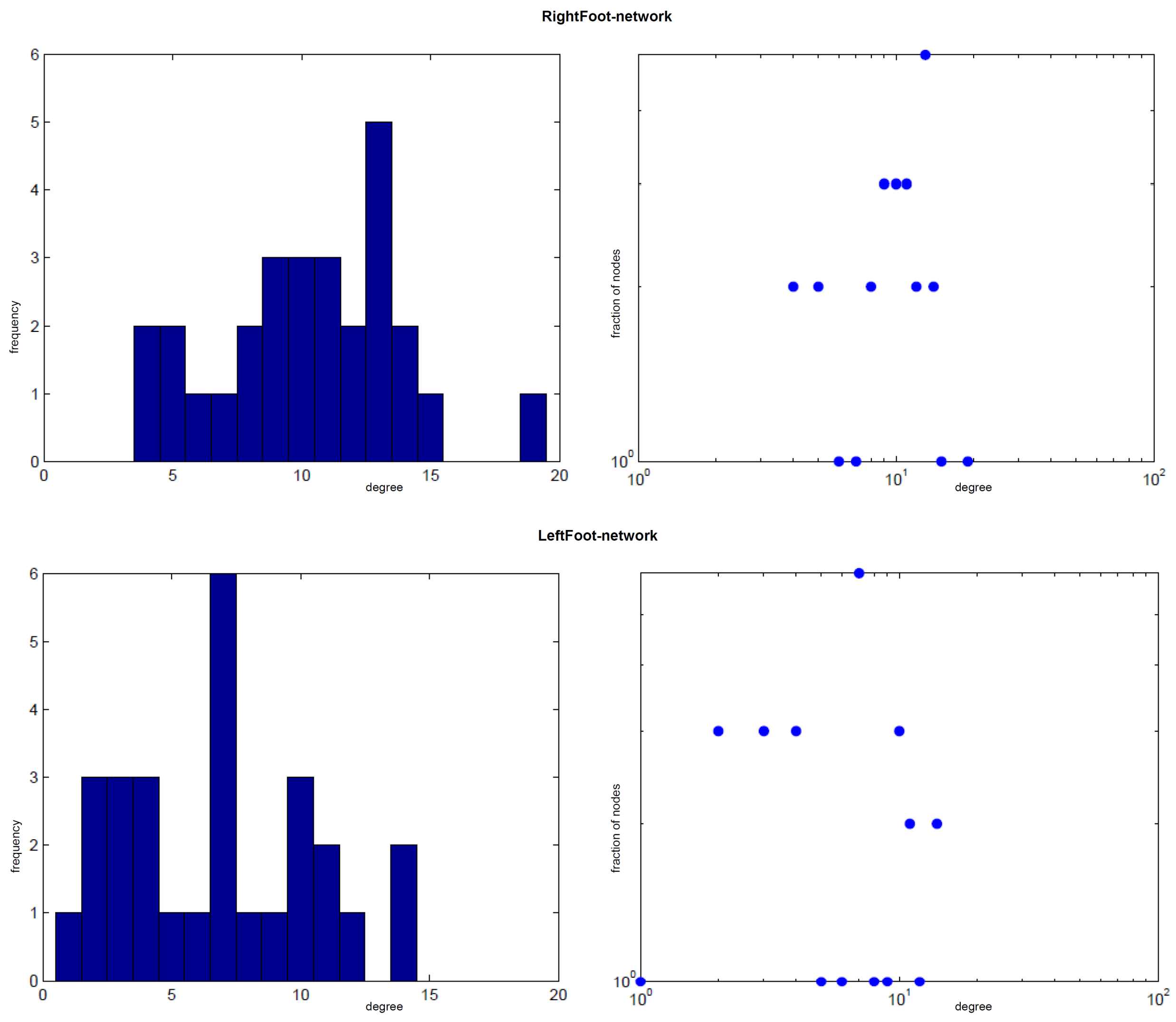


**Supplementary Figure 2. Degree distributions.** Degree distributions shown in histograms and log-log scales. Neither of the two networks follows a heavy-tailed degree distribution, i.e., power-law distribution pattern.

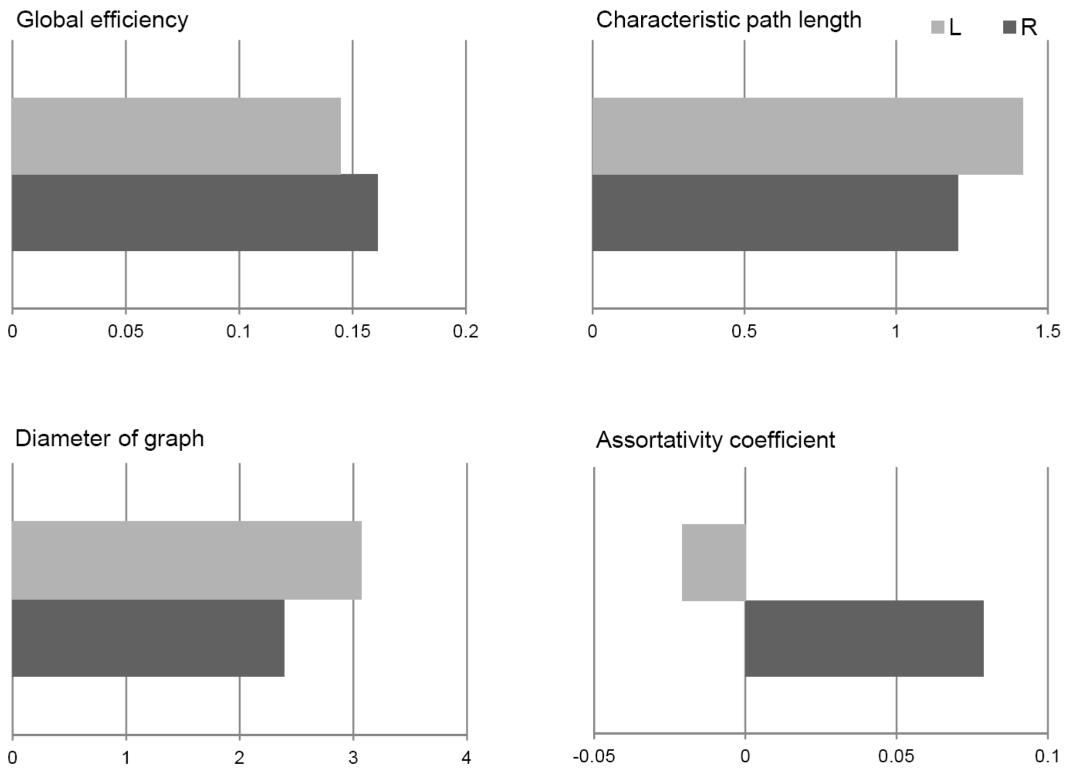


**Supplementary Figure 3. Global properties of the two networks.** Assortativity is the correlation between the degrees of connected nodes. High-degree nodes tend to connect to each other if a network has positive assortativity and is robust to the removal of its highest-degree nodes; by contrast, negative assortativity indicates that high-degree nodes tend to attach to low-degree ones and such networks are more vulnerable (Newman, 2002). The assortativity values of the two networks in this work are lower than those of the resting-state networks reported in another study (Braun et al., 2012) and are only mild deviations from zero. This result indicates that motor control networks during motor execution are less robust to damages on important nodes compared with motor networks during resting state; the same was especially true for the LeftFoot-network in the present study.

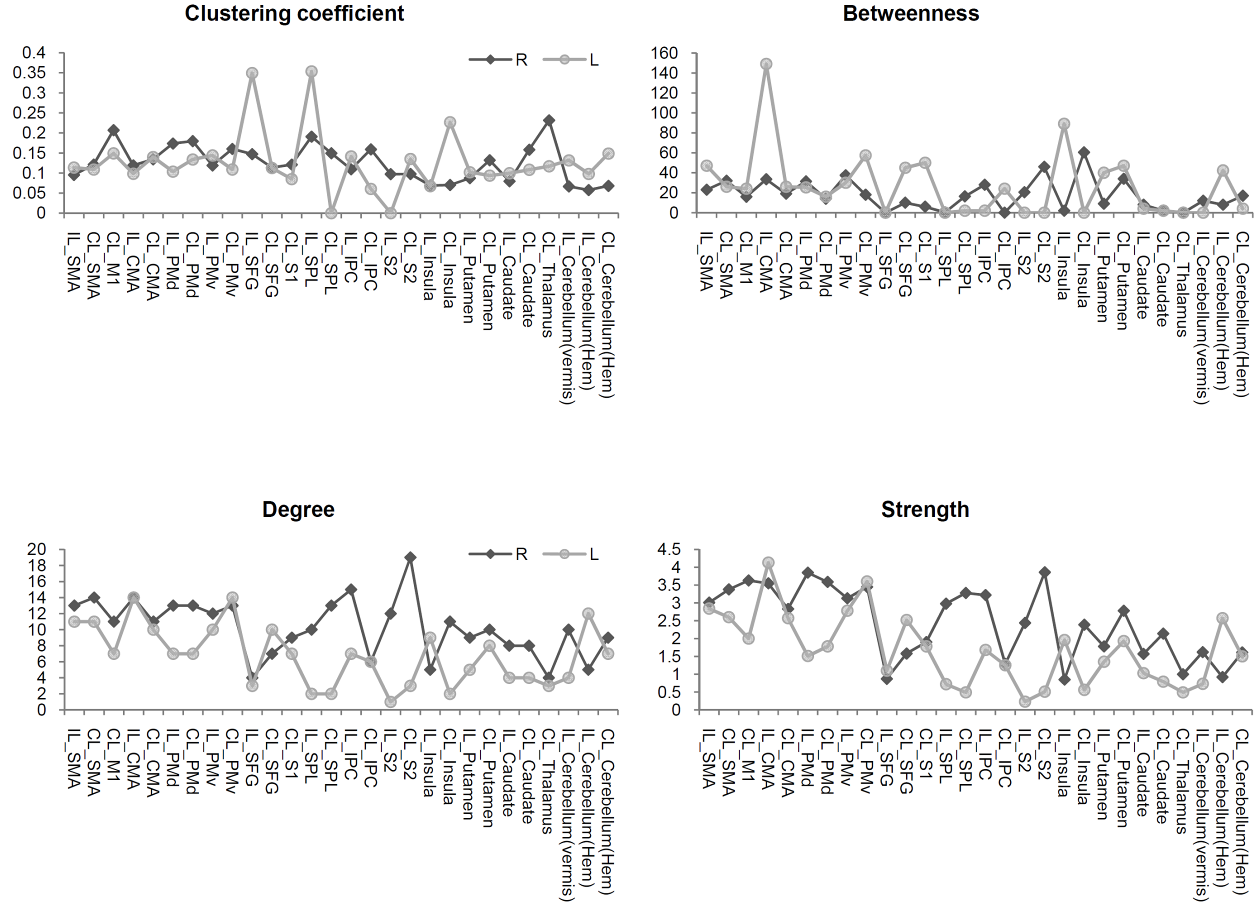


**Supplementary Figure 4. Local properties.** In the LeftFoot-network, the nodes with the highest betweenness values were the ipsilateral CMA and anterior insula; in the RightFoot-network, such nodes were the contralateral anterior insula and S2. In addition, the nodes with the highest degree/strength values moved from two cortical motor areas, namely, CMA and PMv, in the LeftFoot-network to S2 and PMd in the RightFoot-network. Most nodes in the RightFoot-network showed higher degree/strength values than those in the LeftFoot-network; the largest increases occurred in the higher-order cortical sensory areas, namely, SPL, IPC, and S2. In the two networks, the degree/strength values of S1 were approximately at the middle level and close to each other. However, contralateral S2 showed a very low degree/strength value in the LeftFoot-network and a relatively very high value in the RightFoot-network. Ipsilateral S2 showed a similar trend with a lower variation range.

***Hub areas in the motor control network***. Betweenness of a node is defined as the fraction of all the shortest paths in a network that pass through the node and is thus important in controlling information flow across separate parts of the network (Rubinov and Sporns, 2010). Hub nodes are often situated on a high number of shortest paths and consequently have high betweenness values (Boccaletti, et al., 2006). In the LeftFoot-network, the nodes with the highest betweenness values were the ipsilateral CMA and anterior insula, whereas in the RightFoot-network, such nodes were the contralateral anterior insula and S2. The network hubs moved from some motor and cognitive brain areas for the LeftFoot-network to the sensory and cognitive brain areas for the RightFoot-network. Hence, the RightFoot-network requires an increasingly active participation of sensory brain areas.

More specifically, we found that S1 played similar roles in both networks; however, S2 was a hub in the RightFoot-network and not in the LeftFoot-network. This outcome revealed the prominent role of S2 in more skilled movement. Both contralateral S1 and S2 receive inputs from the thalamus, whereas S2 receives inputs from the sources of the kinesthetic, somatic, and motor systems and maintains closer associations with subcortical and cortical motor structures (Karhu and Tesche, 1999; Hinkley, et al., 2007), thereby providing anatomical pathways as the substrate for online sensory prediction and feedback during movement. These characteristics can support S2 with more integrative roles in the RightFoot-network compared with S1.

S1 input to motor cortical areas is an important aspect of sensorimotor integration (Petrof et al., 2015). In this work, the degree/strength values of S1 were proximate in the two networks, thus indicating that S1 played similar roles in both networks. Thus, S1 appeared to be a basic necessity of sensorimotor integration. S2 is also involved in sensory–motor integration (Huttunen et al., 1996; Qi et al., 2002). S2 can be activated during movement (Dobkin et al., 2004; Sahyoun et al., 2004; Kapreli et al., 2006; Kapreli et al., 2007; Newton et al., 2008; Francis et al., 2009), and the activations are modulated by muscle contractions (Lin and Forss, 2002).

Unlike S1, the contralateral S2 was found to have very low degree/strength values in the LeftFoot-network but relatively high values in the RightFoot-network. Ipsilateral S2 showed similar trends with a lower range of variation. Measures of centrality assess the functional influence of individual nodes in a brain network (Rubinov and Sporns, 2011). In a weighted network, the nodal strength integrates values on the number and weights of the edges connecting to a node. Degree (strength as the variant of degree in weighted networks in this work) and betweenness are sensitive measures for hubs (Rubinov and Sporns, 2010). In this study, both measures revealed that contralateral S2 showed high values and was thus a hub in the RightFoot-network, whereas this area showed only very low strength and betweenness values in the LeftFoot-network. Thus, S2 appeared to mainly participate in the RightFoot-network.

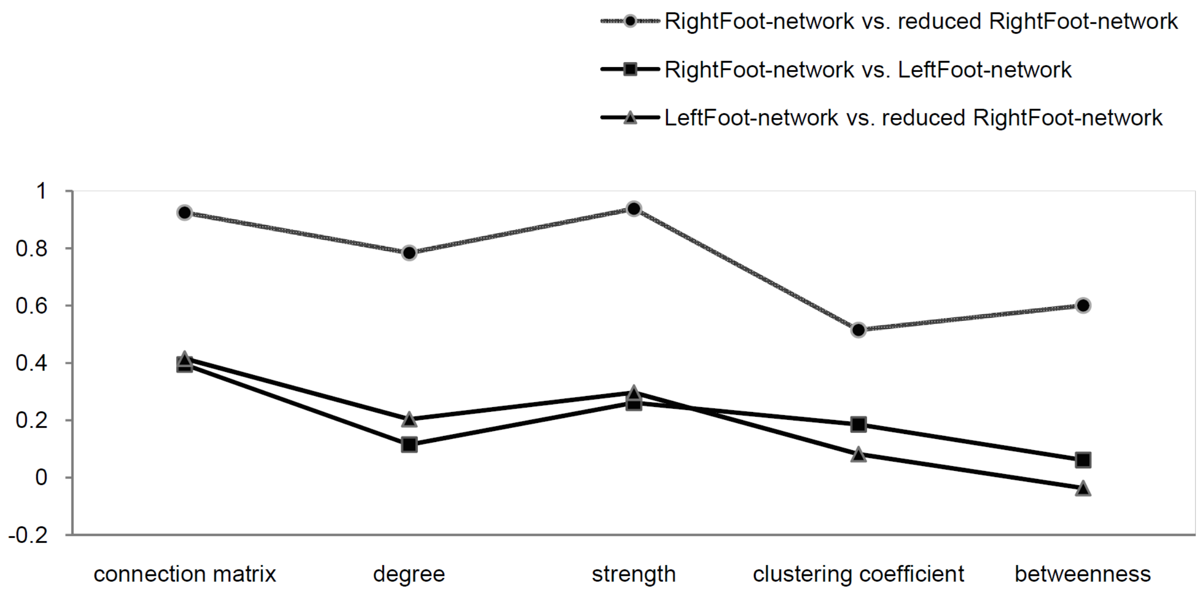


**Supplementary Figure 5. Network properties at the same sparsity.** The correlation coefficients of the connection matrix, nodal degree, strength, betweenness, and clustering coefficient values between each pair of networks. The y-axis shows the correlation values. Different edge numbers or levels of sparsity could be produced at the same threshold; thus, ruling out systematic sparsity differences as the root cause of the detected differences in the topological or organizational properties of networks is difficult (Zalesky et al., 2010). We increased the sparsity of the RightFoot-network to the same level of the LeftFoot-network (that is, reserving its strongest 95 edges on the basis of their Fisher's transformed correlation coefficients and *T* values) and then examined the network measures of this reduced network. Pearson's correlation coefficients of the connection matrix, nodal degree, strength, weighted betweenness, and weighted clustering coefficient values between each pair of networks (i.e., RightFoot-network vs. reduced RightFoot-network, RightFoot-network vs. LeftFoot-network, and LeftFoot-network vs. reduced RightFoot-network) were computed. The highest correlation coefficients always existed between the RightFoot-network and its reduced version, whereas the RightFoot-network vs. LeftFoot-network and LeftFoot-network vs. reduced RightFoot-network showed similar low-level correlations.

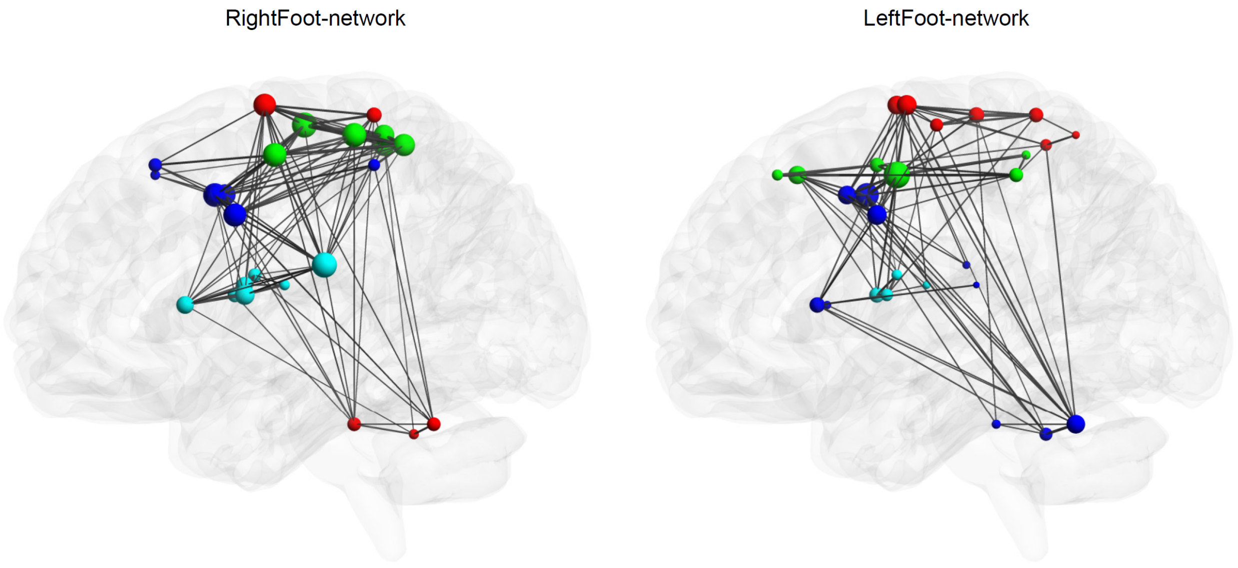


**Supplementary Figure 6. Modules shown in in sagittal view.** Nodes in the same module are indicated in the same color. Nodes are sized according to strength, and edges are sized according to connection weight. Consistent with that in the coronal view, the modules in the sagittal view were also distributed along the cranial–caudal direction. In general, most of the modules were clusters consisting of spatially nearby nodes along such a direction.

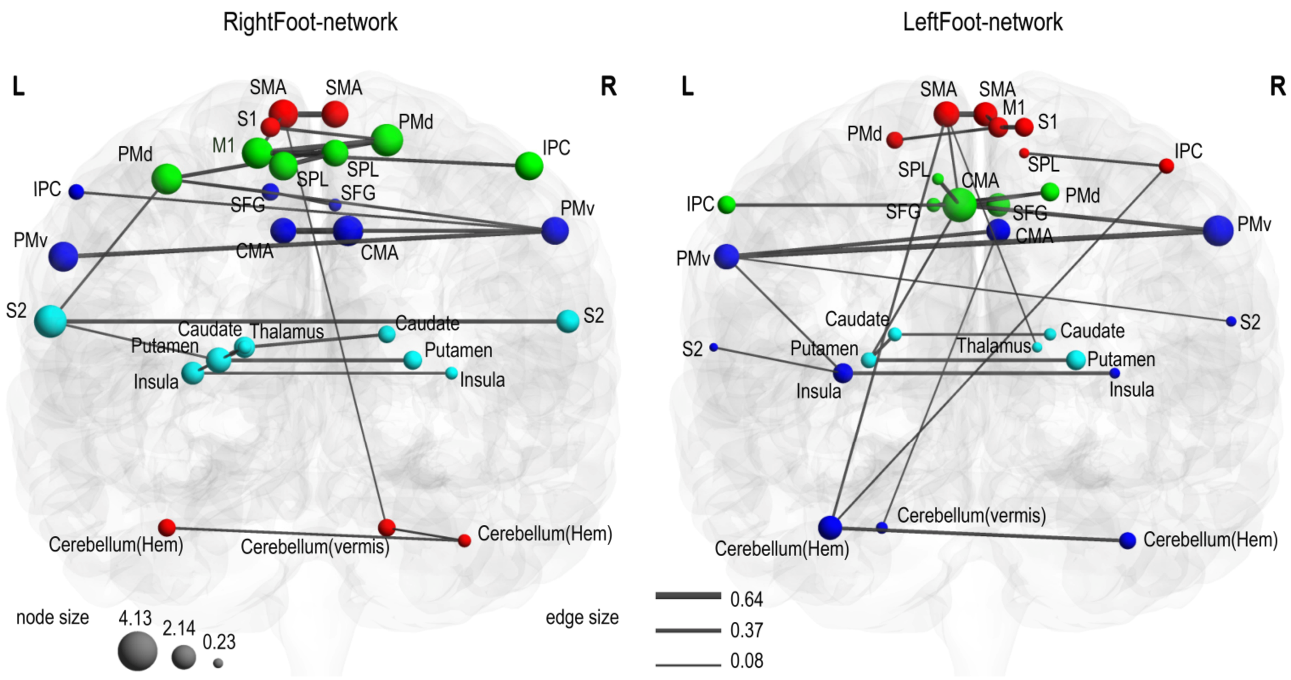


**Supplementary Figure 7. Maximum spanning tree.** The maximum spanning tree was generated first by defining the strongest edge in the network and then adding the strongest edge linking to that dyad. The strongest edge connecting the triad was then added. Edges were continually added until all nodes were linked together (Hidalgo, et al., 2007). This structure can be used to show the "skeleton" of a network. For a functional brain network to complete a task, such a skeleton has an intuitive meaning: these strongest edges constitute the primary route for mainstream information transfer in the system.

## 2.2 Supplementary Tables

### Supplementary Table 1. Peak activations evoked by foot movement.

| Location | Right foot movement | | | | Left foot movement | | | |
|---|---|---|---|---|---|---|---|---|
| | MNI coordinates | | | *T* value | MNI coordinates | | | *T* value |
| L SMA | -6 | -6 | 69 | 12.93 | -6 | -6 | 69 | 12.01 |
| R SMA | 3 | -3 | 69 | 9.67 | 6 | -6 | 69 | 11.54 |
| L M1 | -6 | -27 | 66 | 9.90 | | | | |
| R M1 | | | | | 12 | -33 | 60 | 7.83 |
| L CMA | -3 | -3 | 48 | 7.71 | -6 | 9 | 42 | 7.07 |
| R CMA | 6 | 12 | 42 | 6.31 | 9 | 9 | 42 | 7.46 |
| L PMd | -18 | -15 | 63 | 6.77 | -33 | -9 | 54 | 6.77 |
| R PMd | 18 | 3 | 51 | 6.35 | 18 | -18 | 63 | 7.04 |
| L PMv | -57 | 3 | 36 | 5.70 | -57 | 3 | 36 | 6.31 |
| R PMv | 57 | 6 | 42 | 5.24 | 57 | 6 | 42 | 5.78 |
| L SFG | -9 | 33 | 48 | 4.75 | -9 | 27 | 51 | 4.83 |
| R SFG | 6 | 27 | 48 | 5.61 | 6 | 27 | 48 | 5.75 |
| L S1 | -12 | -45 | 66 | 7.18 | | | | |
| R S1 | | | | | 9 | -39 | 66 | 7.62 |
| L SPL | -8 | -42 | 54 | 4.33 | -6 | -48 | 57 | 4.31 |
| R SPL | 12 | -57 | 60 | 4.08 | 6 | -42 | 60 | 6.83 |
| L IPC | -57 | -39 | 48 | 5.24 | -54 | -39 | 51 | 6.09 |
| R IPC | 45 | -48 | 57 | 4.47 | 51 | -42 | 57 | 6.14 |
| L S2 | -60 | -27 | 15 | 4.52 | -60 | -24 | 21 | 3.99 |
| R S2 | 60 | -24 | 21 | 4.07 | 60 | -24 | 21 | 5.40 |
| L Insula | -30 | 21 | 9 | 4.72 | -27 | 18 | 9 | 5.86 |
| R Insula | 33 | 18 | 9 | 3.80 | 33 | 18 | 9 | 4.59 |
| L Putamen | -24 | 0 | 12 | 4.08 | -21 | 0 | 12 | 4.04 |
| R Putamen | 24 | 3 | 12 | 3.67 | 24 | 3 | 12 | 4.33 |
| L Caudate Nucleus | -18 | -3 | 18 | 3.18 | -15 | 0 | 15 | 2.61 |
| R Caudate Nucleus | 18 | -3 | 18 | 3.36 | 18 | -3 | 18 | 4.12 |
| L Thalamus | -15 | -12 | 15 | 3.58 | | | | |
| R Thalamus | | | | | 15 | -12 | 15 | 3.22 |
| L Cerebellum (vermis) | | | | | -18 | -33 | -27 | 7.04 |
| R Cerebellum (vermis) | 21 | -33 | -27 | 6.50 | | | | |
| L Cerebellum (Hem) | -33 | -57 | -27 | 6.70 | -33 | -57 | -27 | 6.91 |
| R Cerebellum (Hem) | 36 | -48 | -30 | 4.85 | 36 | -51 | -30 | 3.43 |

The spatial locations of peak activations evoked by right or left foot movement were generally mirror symmetric.

CL: contralateral; IL: ipsilateral; R: right, L: left; SMA: the supplementary motor area; M1: the primary motor cortex; CMA: the cingulate motor area; PMd: the dorsal premotor cortex; PMv: the ventral premotor cortex; SFG: the superior frontal gyrus; S1: the primary somatosensory area; SPL: the superior parietal lobule; IPC: the inferior parietal cortex; S2: the secondary somatosensory area; Cerebellum (Hem): cerebellar hemisphere; Cerebellum (vermis): cerebellar vermis.

**Supplementary Table 2. Nodal parameters at different conditions.**

| Parameter / Condition | Degree | Strength | Clustering coefficient | Betweenness centrality |
|---|---|---|---|---|
| RightFoot-network | | | | |
| Mean | 10.29 | 2.45 | 0.13 | 18.00 |
| Standard deviation | 3.60 | 1.00 | 0.05 | 15.30 |
| LeftFoot-network | | | | |
| Mean | 6.79 | 1.68 | 0.13 | 26.86 |
| Standard deviation | 3.74 | 1.00 | 0.08 | 33.27 |
| *P* value | 0.001 | 0.004 | 0.716 | 0.319 |

We performed the Wilcoxon signed rank test to compare local measures because of our lack of understanding of the distribution of most network metrics (Bullmore and Sporns, 2009). Significant statistical differences were still found even with the use of the Bonferroni correction, although the Bonferroni correction method is too conservative and is thus not used here because of the correlations existing among the neighboring samples in the neuroimaging data (Litvak et al., 2011) and the nodes in the networks (Boccaletti, et al., 2006).

**REFERENCES**

Achard, S., Salvador, R., Whitcher, B., Suckling, J., Bullmore, E. (2006). A resilient, low-frequency, small-world human brain functional network with highly connected association cortical hubs. J Neurosci 26:63-72.

Bassett, D.S., Wymbs, N.F., Porter, M.A., Mucha, P.J., Carlson, J.M., Grafton, S.T. (2011). Dynamic reconfiguration of human brain networks during learning. Proc Natl Acad Sci U S A 108:7641-7646. Doi: 10.1073/pnas.1018985108.

Binkofski, F., Kunesch, E., Classen, J., Seitz R,J., Freund, H.J. (2001). Tactile apraxia: unimodal apractic disorder of tactile object exploration associated with parietal lobe lesions. Brain 124:132-144.

Bjornsdotter, M., Loken, L., Olausson, H., Vallbo, A., Wessberg, J. (2009). Somatotopic organization of gentle touch processing in the posterior insular cortex. J Neurosci 29:9314-20.

Boccaletti, S., Latora, V., Moreno, Y., Chavez, M., Hwang, D.-U. (2006). Complex networks: Structure and dynamics. Physics Reports 424:175-308.

Bohlhalter, S., Fretz, C., Weder, B. (2002). Hierarchical versus parallel processing in tactile object recognition: a behavioural-neuroanatomical study of aperceptive tactile agnosia. Brain 125:2537-2548.

Braun, U., Plichta, M.M., Esslinger, C., Sauer, C., Haddad, L., Grimm, O., Mier, D., Mohnke, S., Heinz, A., Erk, S., Walter, H., Seiferth, N., Kirsch, P., Meyer-Lindenberg, A. (2012). Test-retest reliability of resting-state connectivity network characteristics using fMRI and graph theoretical measures. Neuroimage 59:1404-12. doi: 10.1016/j.neuroimage.2011.08.044.

Bullmore, E., Sporns, O. (2009). Complex brain networks: graph theoretical analysis of structural and functional systems. Nat Rev Neurosci 10:186-198. doi: 10.1038/nrn2575.

Dimitrova, A., de Greiff, A., Schoch, B., Gerwig, M., Frings, M., Gizewski, E.R., Timmann, D. (2006). Activation of cerebellar nuclei comparing finger, foot and tongue movements as revealed by fMRI. Brain Res Bull 71:233-241.

Dobkin, B.H., Firestine, A., West, M., Saremi, K., Woods, R. (2004). Ankle dorsiflexion as an fMRI paradigm to assay motor control for walking during rehabilitation. Neuroimage 23:370-381.

Francis, S., Lin, X., Aboushoushah, S., White, T.P., Phillips, M., Bowtell, R., Constantinescu CS (2009). fMRI analysis of active, passive and electrically stimulated ankle dorsiflexion. Neuroimage 44:469-479.

Gao, J.H., Parsons, L.M., Bower, J.M., Xiong, J., Li ,J., Fox, P.T. (1996). Cerebellum implicated in sensory acquisition and discrimination rather than motor control. Science 272:545-547.

Goldberg, I.I., Harel, M., Malach, R. (2006). When the brain loses its self: prefrontal inactivation during sensorimotor processing. Neuron 50:329-339.

Grodd, W., Hulsmann, E., Lotze, M., Wildgruber, D., Erb, M. (2001). Sensorimotor mapping of the human cerebellum: fMRI evidence of somatotopic organization. Hum Brain Mapp 13:55-73.

Hartmann, S., Missimer, J.H., Stoeckel, C., Abela, E., Shah, J., Seitz, R.J., Weder, B.J. (2008). Functional connectivity in tactile object discrimination: a principal component analysis of an event related fMRI-Study. PLoS One 3:e3831. doi: 10.1371/journal.pone.0003831.

Henderson, L.A., Rubin, T.K., Macefield, V.G. (2011). Within-limb somatotopic representation of acute muscle pain in the human contralateral dorsal posterior insula. Hum Brain Mapp 32:1592-601. doi: 10.1002/hbm.21131.

Hidalgo, C. A., Klinger, B., Barabasi, A.-L., Hausmann, R. (2007). The Product Space Conditions the Development of Nations. Science 317:482-487.

Hinkley, L.B., Krubitzer, L.A., Nagarajan, S.S., Disbrow, E.A. (2007). Sensorimotor Integration in S2, PV, and Parietal Rostroventral Areas of the Human Sylvian Fissure. Journal of Neurophysiology 97:1288-1297.

Hotz-Boendermaker, S., Funk, M., Summers, P., Brugger, P., Hepp-Reymond, M.C., Curt, A., Kollias, S.S. (2008). Preservation of motor programs in paraplegics as demonstrated by attempted and imagined foot movements. Neuroimage 39:383-394.

Hua, l.H., Strigo, I.A., Baxter, L.C., Johnson, S.C., Craig, A.D. (2005). Anteroposterior somatotopy of innocuous cooling activation focus in human dorsal posterior insular cortex. Am J Physiol Regul Integr Comp Physiol 289:R319-R325.

Huttunen, J., Wikstrom, H., Korvenoja, A., Seppalainen, A.M., Aronen, H., Ilmoniemi, R.J .(1996). Significance of the second somatosensory cortex in sensorimotor integration: enhancement of sensory responses during finger movements. Neuroreport 7:1009-1012.

Johannsen, P., Christensen, L.O., Sinkjaer, T., Nielsen, J.B. (2001). Cerebral functional anatomy of voluntary contractions of ankle muscles in man. J Physiol 535:397-406.

Kapreli, E., Athanasopoulos, S., Papathanasiou, M., Van Hecke, P., Keleki, D., Peeters, R., Strimpakos, N., Sunaert, S. (2007). Lower limb sensorimotor network: issues of somatotopy and overlap. Cortex 43:219-232.

Kapreli, E., Athanasopoulos, S., Papathanasiou, M., Van Hecke, P., Strimpakos, N., Gouliamos, A., Peeters, R., Sunaert, S. (2006). Lateralization of brain activity during lower limb joints movement. An fMRI study. Neuroimage 32:1709-1721.

Karhu, J., Tesche, C.D. (1999). Simultaneous early processing of sensory input in human primary (SI) and secondary (SII) somatosensory cortices. J Neurophysiol 81:2017-2025.

Koenigs, M., Grafman, J. (2009). Posttraumatic stress disorder: the role of medial prefrontal cortex and amygdala. Neuroscientist 15:540-8. doi: 10.1177/1073858409333072.

Koenigs, M., Huey, E.D., Calamia, M., Raymont, V., Tranel, D., Grafman, J. (2008). Distinct regions of prefrontal cortex mediate resistance and vulnerability to depression. J Neurosci 28:12341-8. doi: 10.1523/JNEUROSCI.2324-08.2008.

Kriegeskorte, N., Simmons, W.K., Bellgowan, P.S., Baker, C.I. (2009). Circular analysis in systems neuroscience: the dangers of double dipping. Nat Neurosci 12:535-540. doi: 10.1038/nn.2303.

Lin, Y.Y., Forss, N. (2002). Functional characterization of human second somatosensory cortex by magnetoencephalography. Behav Brain Res 135:141-145.

Lindner, A., Iyer, A., Kagan, I., Andersen, R.A. (2010). Human posterior parietal cortex plans where to reach and what to avoid. J Neurosci 30:11715-11725. doi: 10.1523/JNEUROSCI.2849-09.2010.

Litvak, V., Mattout, J., Kiebel, S., Phillips, C., Henson, R., Kilner, J., Barnes, G., Oostenveld, R., Daunizeau, J., Flandin, G., Penny, W., Friston, K. (2011). EEG and MEG data analysis in SPM8. Comput Intell Neurosci 2011:852961. doi: 10.1155/2011/852961.

Newman, M.E. (2002). Assortative mixing in networks. Phys Rev Lett 89:208701.

Newton, J.M., Dong, Y., Hidler, J., Plummer-D'Amato, P., Marehbian, J., Albistegui-Dubois, R.M., Woods, R.P., Dobkin, B.H. (2008). Reliable assessment of lower limb motor representations with fMRI: use of a novel MR compatible device for real-time monitoring of ankle, knee and hip torques. Neuroimage 43:136-146.

Petrof, I., Viaene, A.N., Sherman, S.M. (2015). Properties of the primary somatosensory cortex projection to the primary motor cortex in the mouse. J Neurophysiol 113:2400-2407. doi: 10.1152/jn.00949.2014.

Qi, H.X., Lyon, D.C., Kaas, J.H. (2002). Cortical and thalamic connections of the parietal ventral somatosensory area in marmoset monkeys (Callithrix jacchus). J Comp Neurol 443:168-182.

Rubinov, M., Sporns, O. (2010). Complex network measures of brain connectivity: uses and interpretations. Neuroimage 52:1059-1069. doi: 10.1016/j.neuroimage.2009.10.003.

Rubinov, M., Sporns, O. (2011). Weight-conserving characterization of complex functional brain networks. Neuroimage 56:2068-2079. doi: 10.1016/j.neuroimage.2011.03.069.

Sahyoun, C., Floyer-Lea, A., Johansen-Berg, H., Matthews, P.M. (2004). Towards an understanding of gait control: brain activation during the anticipation, preparation and execution of foot movements. Neuroimage 21:568-575.

Stam, C.J., Tewarie, P., Van Dellen, E., van Straaten, E.C., Hillebrand, A., Van Mieghem, P. (2014). The trees and the forest: Characterization of complex brain networks with minimum spanning trees. Int J Psychophysiol 92:129-38. doi: 10.1016/j.ijpsycho.2014.04.001.

Trinastic, J.P., Kautz, S.A., McGregor, K., Gregory, C., Bowden, M., Benjamin, M.B., Kurtzman, M., Chang, Y.L., Conway, T., Crosson, B. (2010). An fMRI study of the differences in brain activity during active ankle dorsiflexion and plantarflexion. Brain Imaging Behav 4:121-131.

van Wijk, B.C., Stam, C.J., Daffertshofer, A. (2010). Comparing brain networks of different size and connectivity density using graph theory. PLoS One 5:e13701. doi: 10.1371/journal.pone.0013701.

Vertes, P.E., Alexander-Bloch, A.F., Gogtay, N., Giedd, J.N., Rapoport, J.L., Bullmore, E.T. (2012). Simple models of human brain functional networks. Proc Natl Acad Sci U S A 109:5868-73. doi: 10.1073/pnas.1111738109.

Viviani, P., Perani, D., Grassi, F., Bettinardi, V., Fazio, F. (1998). Hemispheric asymmetries and bimanual asynchrony in left- and right-handers. Exp Brain Res 120:531-536.

Wang, J., Wang, L., Zang, Y., Yang, H., Tang, H., Gong, Q., Chen, Z., Zhu, C., He, Y. (2009). Parcellation-dependent small-world brain functional networks: a resting-state fMRI study. Hum Brain Mapp 30:1511-1523. doi: 10.1002/hbm.20623.

Wang, L., Yu, C., Chen, H., Qin, W., He, Y., Fan, F., Zhang, Y., Wang, M., Li, K., Zang, Y., Woodward TS, Zhu C (2010). Dynamic functional reorganization of the motor execution network after stroke. Brain 133:1224-1238.

Zalesky, A., Fornito, A., Bullmore, E.T. (2010). Network-based statistic: identifying differences in brain networks. Neuroimage 53:1197-207. doi: 10.1016/j.neuroimage.2010.06.041.

Zalesky,, A., Fornito, A., Harding, I.H., Cocchi, L., Yucel, M., Pantelis, C., Bullmore, E.T. (2010). Whole-brain anatomical networks: Does the choice of nodes matter?. NeuroImage 50:970-983.
